# Clinical Characteristics, Surgical Management, and Outcomes of Borderline Ovarian Tumours: A Retrospective Observational Study from North East London †

**DOI:** 10.3390/jcm14072383

**Published:** 2025-03-30

**Authors:** Kshitij Jamdade, Amal Hashi, Nandita Deo

**Affiliations:** 1 Department of Gynaecological Oncology, Nottingham University Hospitals NHS Trust, City Hospital, Nottingham NG5 1PB, UK; 2 Department of Gynaecological Oncology, Barts Health NHS Trust, Whipps Cross Hospital, London E11 1NR, UK; 3 Department of Obstetrics and Gynaecology, Barts Health NHS Trust, Whipps Cross Hospital, London E11 1NR, UK

**Keywords:** borderline ovarian tumours (BOTs), fertility-sparing surgery, recurrence risk, ultrasound and MRI sensitivity, CA-125 tumour marker, long-term follow-up

## Abstract

**Background:** Borderline ovarian tumours (BOTs) are a unique subset of epithelial ovarian neoplasms characterised by atypical epithelial proliferation without stromal invasion. BOTs are typically diagnosed at an early stage, primarily affect women of reproductive age, and have a favourable prognosis. This study aims to evaluate the clinical characteristics, surgical management, and outcomes of BOTs in a North East London cohort. **Methods:** A retrospective, multicentric analysis was conducted on 69 patients with histologically confirmed BOTs managed between January 2018 and December 2022 across the Barts Health NHS Trust hospitals. Clinical and demographic data, surgical details, histopathological findings, and recurrence rates were analysed. We used descriptive and exploratory statistical methods. **Results:** The mean age at diagnosis was 44 years, with 46.37% under 40, including 18 nulliparous women. Most tumours (91.3%) were FIGO stage I, with mucinous histology predominating (56.52%), followed by serous BOTs (27.53%). Ultrasound and MRI demonstrated 65.45% and 81.5% sensitivities for borderline or malignant features, respectively. Surgical approaches included open surgery (75.4%), laparoscopy (17.4%), and robotic-assisted procedures (2.9%). Fertility-sparing surgery (FSS) was performed in all nulliparous women under 40 years of age. Recurrence occurred in 2 cases, both in patients with prior FSS performed over a decade earlier. **Conclusions:** FSS is a viable option for young women, especially if nulliparous and under the age of 40, with recurrence rates comparable to radical procedures. Most patients were diagnosed early (FIGO I) with excellent prognoses. MRI proved most sensitive for diagnosis, while long-term follow-up with transvaginal ultrasound and CA-125 monitoring is crucial for detecting recurrences.

## 1. Introduction

Borderline ovarian tumours (BOTs) are a subset of epithelial ovarian neoplasms, accounting for 10–15% of all epithelial ovarian cancers. They are characterised by atypical epithelial proliferation without stromal invasion and are typically diagnosed at an early stage, often confined to the ovaries. BOTs primarily affect women during their reproductive years, with one-third of cases occurring before the age of 40. The incidence of BOTs is approximately 4.8 per 100,000 per year in European populations [1].

The risk factors for BOTs are similar to those reported for invasive ovarian tumours. Protective effects of pregnancy and lactation have been consistently observed, with parous women demonstrating a reduced risk of developing BOTs [2]. However, the use of combined oral contraceptives does not confer the same protective benefit for BOTs as it does for invasive ovarian tumours. Additionally, no evidence links BRCA gene mutations to an increased predisposition to BOTs. Interestingly, BOTs have shown familial associations with other malignancies, including colorectal, pancreatic, lung, and bone cancers, as well as leukaemia [3].

Histologically, serous borderline ovarian tumours (sBOTs) represent the majority, accounting for approximately 50–66% of cases, followed by mucinous BOTs (mBOTs). sBOTs are bilateral in 15–25% of cases and may involve peritoneal spread in up to 40%, with a reported relapse rate ranging from 7.8 to 34% [4]. Conversely, mBOTs are typically unilateral, large in size, and have a lower risk of recurrence. However, when recurrence occurs, mBOTs carry a higher risk of progressing to invasive disease [5,6].

The cornerstone of BOT management is surgical staging according to FIGO guidelines. This staging includes bilateral salpingo-oophorectomy, hysterectomy, peritoneal washing, omentectomy, multiple peritoneal biopsies, and, for mucinous BOTs, appendectomy. Given that approximately one-third of BOT cases occur in women under 40, fertility-sparing surgical options, such as ovarian cystectomy or unilateral salpingo-oophorectomy, are often considered.

BOTs have a significantly better prognosis than invasive epithelial ovarian cancers due to the absence of destructive stromal invasion. They are frequently diagnosed at FIGO stage 1, with the disease confined to the ovaries. Current evidence does not support the use of adjuvant chemotherapy or radiotherapy to improve overall survival, and guidelines do not recommend adjuvant treatment for BOTs, regardless of FIGO stage. The primary risk factors for recurrence include the initial FIGO stage and peritoneal implants, particularly invasive ones.

The primary aim of this study was to present our experience in managing BOTs in North East London, and to provide insights into the clinical characteristics, treatment approaches, and outcomes.

## 2. Materials and Methods

We conducted a retrospective, multicentre analysis of patients diagnosed with borderline ovarian tumours (BOTs) between January 2018 and December 2022 across three hospitals within the Barts Health NHS Trust. This retrospective study was designed to analyse trends, associations, and clinical practices within our Trust, aiming to improve patient care and generate hypotheses for future treatment strategies. This study was registered as a clinical audit under the title “Audit of Borderline Ovarian Tumours” with the Barts Health NHS Trust (Audit Registration ID: 13603), and was conducted according to institutional guidelines and governance policies.

We included all patients aged 18 years and older diagnosed with BOTs and receiving treatment and follow-up care within our Trust. Patient cases were identified using the Systematized Nomenclature of Medicine Clinical Terms (SNOMED CT), and data were collected from electronic health records, pathology reports, and surgical notes. The information was compiled into a standardised spreadsheet proforma for analysis. Clinical and demographic variables, including body mass index (BMI), age, menopausal status, tumour localization, size, preoperative CA-125 levels, surgical intervention details, and follow-up data, were analysed from the medical records. There was no loss to follow up.

A specialised pathology team with expertise in ovarian tumours conducted a histopathological evaluation of the tissue specimens, following the World Health Organization (WHO) International Classification for Ovarian Tumours guidelines [7]. Tumour staging was recorded in accordance with the FIGO classification system [8]. Additional information was obtained from private healthcare providers for a small subset of cases to ensure data completeness.

The primary outcomes of this study were the sensitivity of imaging modalities in diagnosing BOTs and the feasibility of fertility-sparing surgery (FSS) in younger women. Secondary outcomes included recurrence rates and postoperative complications.

Categorical variables were analysed using frequency tables, presenting the number and percentage of cases, while quantitative variables were summarised using descriptive statistics. Data were analysed using Microsoft Excel (Version 365, Microsoft Corporation, Redmond, WA, USA).

As a retrospective cohort study utilising de-identified data, this study did not require ethical committee approval and was conducted as per the Declaration of Helsinki.

## 3. Results

Between January 2018 and December 2022, 69 women with a primary diagnosis of histopathologically confirmed borderline ovarian tumours (BOTs) were identified at the Department of Gynaecologic Oncology, Barts Health NHS Trust Hospital. These patients were included in this retrospective study. Demographic characteristics are summarised in Table 1. The mean age at diagnosis was 44 years (range: 20–77). Of the 69 patients, 32 (46.37%) were under 40, including 18 nulliparous women. The mean body mass index (BMI) at diagnosis was 25.5 kg/m^2^ (range: 18–39). More than one-third (39.13%) of women had a normal weight (BMI of 18–24.9 kg/m^2^), compared with 19 patients (27.53%) who were overweight (BMI of 25 to 29.9 kg/m^2^), 21 patients (30.43%) who were obese (BMI of >30 kg/m^2^), and only 2 (2.89%) were underweight (BMI < 18 kg/m^2^).

### 3.1. Clinical Features

Clinical characteristics, including FIGO stage, tumour localisation, CA-125 levels, and histological subtypes, are summarised in Table 2. Mucinous borderline tumours (mBOTs) were the most prevalent subtype, occurring in 39 of 69 cases (56.52%), followed by serous histology in 19 of 69 cases (27.53%). Other histological subtypes included sero-mucinous (7/69; 10.14%) and mucino-intestinal (2/69).

One mucinous BOT exhibited focal intraepithelial carcinoma, but was managed as a borderline tumour. Two serous BOTs demonstrated focal areas of low-grade microinvasion. Most patients (94.2%) had unilateral tumours, with only four cases (5.8%) presenting as bilateral (three serous and one sero-mucinous). The majority of cases were diagnosed at FIGO stage I (91.3%), with three patients (4.34%) at stage II, two (2.89%) at stage III, and one (1.44%) at stage IV.

### 3.2. CA-125 Levels

CA-125 assessment (>35 IU/L) was performed in 66 patients (95.65%). Elevated levels were detected in 38 patients (55.07%), while 28 (40.57%) had normal levels. In three cases, CA-125 testing was not performed. No correlation was observed between elevated CA-125 levels and the FIGO stage.

### 3.3. Imaging Findings

Ultrasound was performed in 55 patients (79.7%). Thirty-five of the fifty-five women (64%) who underwent ultrasound had findings suggestive of either borderline or malignant tumour, whilst the remaining 36% were thought to be benign on USS. Among these, six showed bilateral cysts. However, intra-op findings and histology confirmed bilateral cysts in only 4 cases. Specific findings included: Twelve patients (17%) were incidentally diagnosed with ovarian cysts during investigations for non-gynaecological symptoms. MRI was conducted in 54 patients (78.3%). Overall, 44 of 54 patients (81.5%) had MRI findings suggestive of borderline or malignant tumours, demonstrating a higher sensitivity (81.5%) compared with ultrasound (65.45%) in detecting these features. CT scans were performed in 54 patients (78.3%), primarily for staging and postoperative evaluation.

### 3.4. Surgical Management

All 69 patients were reviewed at the Gynaecologic Oncology Multidisciplinary Team (MDT) meetings to guide clinical management. Among them, 54 underwent open surgery, 12 underwent laparoscopy, 2 had robotic surgery, and 1 patient underwent an ovarian cystectomy during a caesarean section. All 18 nulliparous patients under 40 years of age underwent FSS.

Most of the surgeries were uneventful. However, we noted some surgical complications, including haemorrhage in 3 cases (2700 mL, 2000 mL, and 1100 mL, respectively) and tumour spillage (classified as FIGO stage IC1) in 10 cases. Tumour spillage happened in 8 cases operated with laparotomy and 2 cases operated laparoscopically.

Frozen section analysis was performed in 17 patients during surgery to aid intraoperative diagnosis. Among these, 16 were confirmed to have BOT, one of which was reported as BOT with invasion not ruled out, and the remaining one had calcifications only. Out of the eventual total of 39 mBOTs, appendectomy was performed in 16 patients, either preoperatively diagnosed or suspected intraoperatively. None of the patients underwent lymphadenectomy.

We performed repeat surgeries in nine cases. Among these, six were completion surgeries, including salpingo-oophorectomy, omentectomy, and hysterectomy. One patient presented with vault dehiscence following a hysterectomy and bilateral salpingo-oophorectomy and required vault dehiscence repair. Another patient, who had undergone bowel resection, anastomosis, and a defunctioning ileostomy as part of pelvic clearance for BOT, underwent ileostomy reversal. Additionally, one patient with mBOT, who initially presented with a large abdominal mass, requested excess skin removal after the tumour excision. The plastic surgery team performed this procedure.

### 3.5. Follow-Up

We followed our patients with transvaginal ultrasonography (TVS) and serum CA-125 levels to assess recurrence risk, especially those who had FSS. Other patients were followed up mainly with CA-125 and clinical examination. Eleven patients underwent Oesophagogastroduodenoscopy (OGD) as part of an MDT-recommended workup, especially those with mucinous histology. All of these were negative for gastrointestinal involvement.

In our study, two patients presented with a recurrence of BOT. One of them had salpingo-oophorectomy 10 years back for stage 1c. The other one had ovarian cystectomy for stage 1 as part of FSS 10 years back. Notably, her primary surgery was conducted outside the UK, and details regarding intraoperative spillage were unavailable.

### 3.6. Miscellaneous Findings

Among the 69 cases, one serous BOT exhibited focal microinvasion (stage II) and one mucinous BOT displayed focal intraepithelial carcinoma. BOTs were identified incidentally in 6% of cases following histological evaluation of surgeries initially performed for suspected benign pathology.

## 4. Discussion

This retrospective study evaluated 69 women with borderline ovarian tumours (BOTs) over a 5-year follow-up period. Among these, 67 cases involved a primary diagnosis of BOT, while 2 cases had a prior history of BOT with FSS performed 10 years earlier. These findings reaffirm the generally favourable prognosis associated with BOTs. As reported in the current literature, the risk of recurrence is typically low [9,10]. However, it is well-documented that BOTs may recur even 15–20 years after initial treatment, highlighting the importance of long-term follow-up to ensure early detection and management of any delayed recurrences. Indeed, our results from two patients confirmed this, but sadly, we do not have an incidence rate.

BOTs are generally diagnosed at a younger age compared with invasive ovarian cancer, with a reported age range of 28–62 years, approximately a decade earlier than invasive ovarian malignancies. This trend is consistent with our study, where the median age at diagnosis was 44 years [1,2,11,12,13]. The studies by Pirimoglu et al., Ji et al., and Desfeux et al. reported a lower incidence of BOTs among postmenopausal women [14,15,16].

Preoperative assessment of the CA-125 tumour marker remains essential for diagnosing and monitoring BOTs. In this study, serum CA-125 levels were measured preoperatively in 95.65% of patients, with elevated levels observed in over half of the cases. Wong et al. reported elevated CA-125 levels in 39% of patients [17], while Ren et al. observed a higher proportion of elevated CA-125 levels (62%) and demonstrated a correlation between elevated serum CA-125 levels and advanced-stage BOT [13]. However, our findings showed that 91.3% of women with elevated CA-125 levels were diagnosed at FIGO stage 1.

Ayhan et al. examined the relationship between elevated CA-125, CA 199, and CEA levels in serous and mucinous BOTs. They found that CA-125 was elevated in serous BOT, whereas CA 199 and CEA levels were significantly higher in mucinous BOT compared with serous BOT [18]. However, our findings showed elevated CA-125 levels in 57.89% of serous, 58.97% of mucinous, and 14.28% of sero-mucinous histology.

Most BOTs are of serous or mucinous subtypes, with rare histological types, including endometrioid, clear cell, and transitional cell (Brenner) tumours [19,20,21]. In our study, the distribution of histological subtypes differed from those reported in the literature, with 27.53% of cases being serous and 56.52% being mucinous BOT. Recent studies report a histopathological distribution of 55–65% serous and 34–45% mucinous tumours [3,22,23]. These findings, which differ from those reported in the literature, may be explained by the limited sample size of our study population and the uneven distribution of events.

We observed bilateral tumours in 5.8% of cases, lower than the reported incidence of 10–40% [24]. This finding could be because we had more mBOTs than sBOTs, and the incidence of bilateral cysts in mBOTs is lower. While Morris et al. and Hog et al. found no correlation between microinvasion and overall prognosis [25,26], Ren et al. reported a 39% recurrence rate in BOTs with microinvasion compared with 10% in those without microinvasive features [13]. In our study, one patient with serous histology (stage 2) exhibited focal microinvasion, and another with mucinous BOT showed focal intraepithelial mucinous carcinoma. None of our patients had a recurrence of the disease during follow-up. The average follow-up duration was 50.46 months (Range: 29–82), with no cases of follow-up loss.

In sBOTs, specific histological subtypes, such as the micropapillary variant, are associated with a higher risk of recurrence and progression to low-grade serous carcinoma (LGSC) than conventional sBOTs [27]. However, Du Bois et al. [28] found no significant correlation between disease recurrence and features like microinvasion or the micropapillary growth pattern. Including histologic subtypes in evaluating sBOT cases may offer further prognostic information and improve the clinical significance of the results. Histopathological characteristics, including microinvasion and micropapillary architecture, may influence recurrence risk. Microinvasion, defined in the 2014 WHO classification [19], refers to stromal cell clusters with a large eosinophilic cytoplasm, resembling epithelial cells, extending up to 5 mm. The micropapillary variant, recognised as a distinct S-BOT subtype, constitutes 5–15% of cases and was initially termed “non-invasive LGSC” by Kurman [29]. This variant is characterised by cribriform epithelial lining, fibrovascular papillae, or elongated filiform micropapillae (length-to-width ratio 5:1), with cribriform growth exceeding 5 mm. Newer diagnostic criteria highlight greater nuclear atypia, including a high nucleus-to-cytoplasm ratio, prominent nucleoli, and rounded cells. Kurman suggested that the micropapillary variant may represent an intermediate stage between typical S-BOT and LGSC [30]. The prognostic significance of micropapillary patterns and microinvasion remains controversial, with some studies identifying them as risk factors and others reporting no impact on survival or disease-free outcomes, except when associated with invasive peritoneal implants [19].

Standard surgical guidelines recommend complete staging, including bilateral salpingo-oophorectomy, hysterectomy, peritoneal biopsies, omentectomy, and peritoneal washing with cytology [31]. However, there is an increasing role of cystectomy in patients needing FSS and most of the recurrence occurs within the same ovary [32,33].

For mucinous tumours, appendectomy is also advised [34]. Surgery may be performed via laparoscopy or laparotomy. The ROBOT multicentre study found no significant impact of the surgical approach on recurrence rates or overall survival [28]. However, cyst rupture (33.9% vs. 12.4%) and incomplete staging were more common with laparoscopy [35]. In our case series, 10 patients experienced cyst rupture or spillage, with 8 cases occurring during open surgery and 2 during laparoscopic procedures. The higher incidence of cyst rupture during laparotomy can be explained by our cohort’s higher incidence of mBOTs.

The risk of reoperation and recurrence must be carefully considered, especially in FSS, which are increasingly offered to women under 40 years of age. Conservative management of BOTs, including the preservation of at least one ovary and the uterus, has been associated with recurrence rates of 10–20%, compared with 5% with radical surgery, without an increase in mortality [28,36,37,38,39,40]. In our cohort, 32 patients were under 40, of which 18 nulliparous women underwent FSS.

Trillsch et al. identified incomplete staging as an adverse prognostic factor, particularly for serous BOTs, with omentectomy having the most significant prognostic impact in both unadjusted and multivariate analyses [41]. In this study, both recurrent cases involved patients who had undergone FSS a decade earlier and were diagnosed at an early stage (FIGO stage I). Although lymph node involvement has been reported in up to 29% of cases, recurrence, and survival rates are similar regardless of lymph node status [16,42,43]. No lymphadenectomies were performed in our group of patients.

### Strengths and Limitations of the Study

While this study provides valuable insights into the clinical characteristics, surgical management, and short-term outcomes of borderline ovarian tumours, we must acknowledge several limitations. The study included only 69 patients, which limits the statistical power and generalisability of the findings. A larger sample size would enhance the reliability and strength of the conclusions. The study was conducted at a single institution, which may introduce selection bias and limit the applicability of results to broader populations. The length of follow-up may not be sufficient to capture late recurrences or long-term survival outcomes comprehensively. As with any retrospective study, missing or incomplete records could impact the accuracy and reliability of the reported findings. The study does not include molecular profiling, which could provide additional insights into the biological behaviour of borderline ovarian tumours and their prognosis. Differences in surgeon experience, institutional protocols, and patient preferences could have influenced the management and outcomes, introducing potential confounding factors. The study lacks a comparative control group, which limits the ability to directly assess treatment efficacy or compare outcomes with other management strategies. Given the retrospective nature, there may be underreporting of recurrence due to loss to follow-up or incomplete medical records. However, all patients remained in follow-up, minimising this limitation.

Addressing these limitations in future research, such as prospective multicentre studies with larger cohorts and standardised treatment protocols, will help validate and strengthen these findings.

## 5. Conclusions

This retrospective study provides valuable insights into the clinical characteristics, management, and outcomes of borderline ovarian tumours (BOTs). The majority of cases were diagnosed at FIGO stage I, with mucinous histology being the most prevalent subtype. MRI demonstrated the highest sensitivity in detecting BOT features, reinforcing its role in preoperative assessment. Although CA-125 was elevated in over half of the patients, no correlation with tumour stage was observed, limiting its utility for staging.

Surgical management was predominantly by laparotomy, with FSS offered to all eligible nulliparous women under 40. All women under 40 who are nulliparous can be offered FSS. While most patients achieved excellent prognoses following surgical intervention, tumour spillage remained a concern, occurring more frequently in laparotomy than laparoscopy. Long-term follow-up is essential, particularly for those undergoing FSS, relying on transvaginal ultrasound and CA-125 monitoring to detect recurrences. These findings contribute to optimising BOT management and underscore the need for ongoing research into surgical techniques and surveillance strategies.

## Figures and Tables

**Table 1:** Demographics.

Characteristics	N = 69
Age	44
Median age (range)	20–77
Age groups (n (%))	
<40 years	32 (46.37%)
≥40 years	37 (53.62%)
Parity (n (%))	
Nulliparous	35 (50.72%)
Parous	34 (49.27%)
Body mass index (kg/m^2^)	
Median BMI (range)	25.5 (18–39)
BMI groups (n (%))	
Underweight (<18)	2 (2.89%)
Normal (18–24.9)	27 (39.13%)
Overweight (25–29.9)	19 (27.53%)
Obesity (>30)	21 (30.43%)

**Table 2:** Tumour characteristics by histological subtypes of borderline ovarian tumours.

Tumour Characteristics				Tumour Sub-Types		
		Serous N = 19 (27.53%)	Mucinous N = 39 (56.52%)	Sero-Mucinous N = 7 (10.14%)	Mucino-Intestinal N = 2 (2.89%)	Other N = 2 (2.89%)
FIGO	I	13 (68.42)	39 (100%)	7 (100%)	2 (100%)	2 (100%)
	II	3 (15.78%)	0 (0.0%)	0 (0.0%)	0 (0.0%)	0 (0.0%)
	III	2 (10.52%	0 (0.0%)	0 (0.0%)	0 (0.0%)	0 (0.0%)
	IV	1 (5.26%)	0 (0.0%)	0 (0.0%)	0 (0.0%)	0 (0.0%)
Location	Unilateral	16 (84.21%)	39 (100%)	6 (85.71%)	2 (100%)	2 (100%)
	Bilateral	3 (15.78%)	0 (0.0%)	1 (14.28%)	0 (0.0%)	0 (0.0%)
CA-125	<35	7 (36.84%)	15 (38.46%)	5 (71.42%)	0 (0.0%)	1 (50.0%)
	>35	11 (57.89%)	23 (58.97%)	1 (14.28%)	2 (100%)	1 (50.0%)
	Missing	1 (5.26%)	1 (2.56%)	1 (14.28%)	0 (0.0%)	0 (0.0%)
Microinvasion		2 (10.52%)	NA	NA	NA	NA
Focal intraepithelial Carcinoma		NA	1 (2.56%)	NA	NA	NA

## Data Availability

The data presented in this study are available upon request from the corresponding author due to privacy and trust policy.
